# Changes in IL-2 and IL-10 during Chronic Administration of Isoniazid, Nevirapine, and Paracetamol in Rats

**DOI:** 10.1155/2016/3094783

**Published:** 2016-11-20

**Authors:** Zanelle Bekker, Andrew Walubo, Jan B. Du Plessis

**Affiliations:** Department of Pharmacology, University of the Free State, P.O. Box 339, Bloemfontein 9300, South Africa

## Abstract

The aim of this study was to illustrate the initial subclinical drug-induced liver injury and the associated adaptive immune response by monitoring for the changes in plasma IL-2, IL-10, and some cytochrome P450 activity during chronic administration of nevirapine (NVP), isoniazid (INH), and paracetamol (PAR) in rats without clinical hepatotoxicity. Male Sprague-Dawley (SD) rats were divided into four groups (saline (S), NVP, INH, and PAR) of 25 animals each. The drugs were administered daily for 42 days at therapeutic doses (NVP 200 mg/kg, PAR 500 mg/kg, and INH 20 mg/kg) to the respective groups by oral gavage and five rats per group were sacrificed weekly. All the three drugs induced a subclinical liver injury in the first 2-3 weeks followed by healing, indicating adaption. The liver injury was pathologically similar and was associated with immune stimulation and increased cytochrome P450 activity. NVP- and PAR-induced liver injury lasted up to 14 days while that for INH lasted for 28 days. NVP-induced liver injury was associated with increased IL-2, CD4 count, and CYP3A2 activity, followed by increased IL-10 during the healing phase. In conclusion, the initial drug-induced subclinical liver injury, its spontaneous healing, and the associated adaptive immune response have been demonstrated.

## 1. Introduction

Drug-induced liver injury is a major contributor to adverse drug reactions that has restricted the use of efficacious drugs such as isoniazid (INH) and nevirapine (NVP), while paracetamol (PAR) overdose is associated with fatal drug-induced liver injury. Although several mechanisms regarding INH, NVP, and PAR-induced hepatotoxicity have been postulated, the immune system has been implicated as a mediator and major determinant for progression of the liver injury [1–4]. It was proposed that metabolic activation of these drugs leads to the formation of reactive metabolites, which attack cellular proteins and result in the formation of metabolite-protein adducts, some of which are antigenic [5–9]. As a result, the immune system is activated and starts a process to eliminate hepatocytes expressing these immunogenic adducts [10–13]. It was then explained that most patients do not develop hepatotoxicity because their counter mechanisms are able to efficiently eliminate the antigenic adducts and/or to counter the proinflammatory response [14–17]. The elimination process is mediated by proinflammatory cytokines such as tumour necrosis factor-alpha (TNF-*α*), interleukin-1beta (IL-1*β*), IL-2, and IL-4, while the counter mechanisms are mediated by anti-inflammatory cytokines such as IL-6, IL-10, and IL-13. However, in a few individuals, there is failure to counter the proinflammatory response, and this leads to progressive destruction of hepatocytes and overt drug-induced hepatotoxicity ensues [16, 17]. Indeed, several reports have described increased levels of some proinflammatory cytokines during hepatotoxicity by INH [18], PAR [19], and NVP [20].

Unfortunately, this explanation of initial hepatic injury and recovery, though plausible, has never been illustrated. It was envisaged that an experimental illustration of the initial hepatic injury and recovery is critical to promoting further research on preventive strategies for drug-induced hepatotoxicity such as the development of biomarkers for early prediction of patients who are likely to progress to overt hepatotoxicity. Therefore, the aim of this study was to illustrate the initial drug-induced subclinical liver injury and associated changes in the plasma profiles of IL-2, a proinflammatory cytokine, and IL-10, an anti-inflammatory cytokine, as well as the activity of the relevant cytochrome P450 isoforms (CYP3A2, CYP1A2, and CYP2E1) during chronic administration of NVP, INH, and PAR in rats.

## 2. Methods

### 2.1. Materials and Reagents

NVP (Viramune) oral suspension of 50 mg/5 ml and tablets 200 mg (Boehringer Ingelheim Pharmaceuticals, Inc., Ridgefield, CT, USA) were purchased from a local pharmacy while INH and PAR were from Sigma-Aldrich Inc. (St. Louis, MO, USA). ELISA kits for rat interleukin-2 (IL-2) and interleukin-10 (IL-10) were purchased from RayBio (RayBiotech Inc., Norcross, GA, USA) and Invitrogen (Invitrogen Corporation, Camarillo, CA, USA), respectively.

### 2.2. Animal Care

Ethical approval was obtained from the Interfaculty Animal Ethics Committee of the University of the Free State. Male Sprague-Dawley (SD) rats weighing 200–250 g were used. Animals were housed at the Animal House of the University of the Free State, where they were fed and looked after by qualified staff. Standard rat chow and water were available to the animals* ad libitum*. Drug administration was done in a side room at the Animal House, and animals were inspected for skin lesions and other visible adverse events every day.

### 2.3. Experimental Design

Rats were divided into four groups of 25 animals each, that is, saline (S; control group), NVP, INH, and PAR. The drugs were administered daily at therapeutic doses to the respective groups by oral gavage (S, NVP 200 mg/kg, INH 20 mg/kg, and PARA 500 mg/kg), and five rats per group were sacrificed after 2, 7, 14, 28, and 42 days of treatment. The respective doses were used in previous studies to produce a concentration range similar to the therapeutic range in humans: NVP [20], INH, and PAR [21]. This because small animals have a higher metabolic rate than humans. The doses were adjusted according to weight every week. A separate group of five rats were not treated with any drug and were used for baseline data.

### 2.4. Blood Collection and Surgical Procedure

Under isoflurane anaesthesia, blood (8 ml) was drawn by cardiac puncture and immediately aliquoted to the appropriate test tubes. Thereafter, the abdomen was opened through a vertical incision to expose the liver. A piece of liver (10 g) was cut and stored in 10% formalin and sent for histopathology. The remainder of the liver was quickly dissected out, washed in a 1.5% potassium chloride solution, frozen with liquid nitrogen, and stored at −85°C. Thereafter, the animals were sacrificed by exsanguination while being still under anaesthesia. Blood or plasma was analysed for full blood count, CD4 and CD8 counts, cytokines, drug concentrations, and liver and renal function.

The liver and renal function tests were analysed in the Toxicology Laboratory of the Department of Pharmacology, while the full blood count (FBC) was done at the National Health Laboratory Service (NHLS), Bloemfontein, South Africa. The CD4 and CD8 counts were analysed by cytometry at the Department of Haematology and Cell Biology, University of the Free State, and histopathology of the livers was performed and reported by an independent veterinary pathologist (Idexx Laboratories, Johannesburg, South Africa).

### 2.5. Analysis of Cytokines

The serum cytokines (IL-2 and IL-10) were detected by enzyme linked immune-assay (ELISA) technique using a Multiskan Ascent UV spectrophotometer with a 96-well microplate reader (Thermo Electric Corp., Shanghai, China), according to the manufacturer's instructions. The ELISA well plates were already precoated with the specific capture antibody of the respective cytokine to be analysed. The appropriate volumes of the incubation buffer, respective standards, and samples as well as the biotin-conjugate secondary antibody were added to the relevant wells and then incubated for 2 hours at room temperature. Thereafter, streptavidin-horseradish peroxidase was added and the plate was incubated for 30 minutes at room temperature. Lastly, a chromogen substrate was added and the plate was further incubated in the dark for 30 minutes at room temperature. The reaction was stopped by addition of acid and absorption was measured at 450 nm with a microplate reader. A standard curve was prepared for each cytokine in pg/ml, and for IL-2, the standard curve concentration ranged from 23 to 1500 pg/ml, while for IL-10 it was 15–1000 pg/ml.

### 2.6. Drug Analysis

The plasma concentrations of NVP, INH, and PAR were measured using a high performance liquid chromatography (HPLC) method for simultaneous determination of the three drugs. Briefly, to 100 *μ*L of plasma, 20 *μ*L of sulfapyridine (internal standard) was added and vortexed for 15 seconds. Thereafter, 50 *μ*L of 15% zinc sulphate and 50 *μ*L of methanol were added to precipitate the proteins. The sample was vortexed for 30 seconds and then centrifuged at 7026 ×g for 15 minutes. The supernatant was further purified by solid phase extraction; a C_18_ solid phase extraction cartridge (1 ml) was conditioned with 2 ml HPLC grade methanol, 2 ml deionised water, and 2 ml 0.05 M potassium phosphate buffer (pH 4.5), respectively. The supernatant was then placed on the column and allowed to elute. Thereafter, the column was washed with 100 *μ*L of potassium phosphate buffer. The compounds were eluted with 100 *μ*L HPLC grade acetonitrile followed by 100 *μ*L of methanol of which 50 *μ*L was injected in the HPLC. The analyte was eluted with a gradient mobile phase of 0.06% trifluoroacetic acid (A) and acetonitrile (B) over a C_18_ (4.60 × 250 mm) 5 *μ*m analytical column at 1 ml/min and was detected by UV at 260 nm. The respective retention times for INH, PAR, IS, and NVP were 3.1, 9.8, 10.4, and 11.6 minutes. The 5-day calibration curves for INH (1–10 *μ*g/ml), NVP (1–10 *μ*g/ml), and PAR (1–20 *μ*g/ml) were linear with correlation coefficients (*r* ^2^) of 0.9954, 0.9968, and 0.9997 and accuracy above 98%, 94%, and 97%, respectively.

### 2.7. Microsomal Preparation and CYP450 Activity

A small piece (1-2 g) of liver was homogenised and microsomes were prepared by differential density ultracentrifugation, and the microsomal pellets were resuspended in 0.1 M potassium phosphate buffer containing 20% glycerol and stored at −85°C until use as reported by Von Moltke et al. (1993) [22]. Total protein concentration was determined by the Biuret method, while the microsomal enzyme activities for CYP3A2, CYP2E1, and CYP1A2 were measured by HPLC using a midazolam, chlorzoxazone, and 7-ethoxyresorufin as the respective enzyme markers.

#### 2.7.1. CYP3A2 Activity

This was a modification of the procedure described by Von Moltke (1996) [23]. To 260 *μ*L of 0.1 M sodium phosphate buffer (pH 7.4) were added final concentrations of 0.8 mg/ml microsomal protein, 4 mM magnesium chloride, and 61.3 nmol midazolam. After preincubation for 5 minutes at 37°C, the reaction was started by the addition of the NADP regenerating system and incubated for 10 minutes at 37°C, after which the reaction was stopped by the addition of 250 ml of cold HPLC grade acetonitrile and 50 *μ*L carbamazepine (internal standard). Thereafter, the sample was alkalinised with sodium hydroxide and extracted with diethyl ether by liquid-liquid extraction. The organic phase evaporated under nitrogen, and the residue was reconstituted with 150 *μ*L of mobile phase of which 100 *μ*L was injected into the HPLC for analysis of midazolam, 1-hydroxymidazolam, and the internal standard. The compounds were analysed on a reversed phase Ultrasphere C18 analytical column (5 *μ*m, 250 mm × 4.6 mm ID) (Beckman, USA) with a mobile phase of 45% acetonitrile in sodium acetate buffer (pH 4.0) at 1 ml/min, and detection was by UV at 220 nm.

#### 2.7.2. CYP2E1 Activity

This was a modification of the procedure described by Peter et al. (1990) [24]. To 250 *μ*L of 0.1 M sodium phosphate buffer (pH 7.4) were added final concentrations of 0.2 mg/ml microsomal protein and 505 nmol chlorzoxazone. After preincubation for 5 minutes at 37°C, the reaction was started by the addition of the NADP regenerating system and was incubated for 10 minutes at 37°C after which the reaction was stopped by the addition of 40 *μ*L of 0.1 M hydrochloric acid and 15 *μ*L paracetamol (internal standard). Thereafter, the samples were analysed by HPLC for chlorzoxazone and its metabolite, 6-hydroxychlorzoxazone. The sample was purified by solid phase extraction on a 6 cc Bond elut C18 column catridge, after which 30 *μ*L of the eluent was analysed on a RP Ultrasphere C18 analytical column (5 *μ*m, 250 mm × 4.6 mm ID) (Beckman, USA) using a gradient solvent system for a mobile phase consisting of acetonitrile (solvent A) and phosphate buffer (pH 4.5; solvent B) over 15 min. Detection was by UV at 280 nm.

#### 2.7.3. CYP1A2 Activity

This was a modification of the procedure described by Burke and Mayer (1974) [25]. To 210 *μ*L of 0.1 M HEPES potassium salt buffer (pH 7.4) were added final concentrations of 0.2 mg/ml microsomal protein, 60 *μ*M EDTA, 5 mM magnesium sulphate, and 20 nmol ethoxyresorufin. After preincubation for 5 minutes at 37°C, the reaction was started by the addition of the NADP regenerating system and incubated for 10 minutes at 37°C after which the reaction was stopped by the addition of 2.5 ml cold acetonitrile. The sample was transferred to a quarts cuvette and detection was read by fluorescence at wavelengths for resorufin of excitation of 560 nm and emission of 585 nm.

### 2.8. Data Analysis

Data was analysed by nonparametric methods using the GraphPad Instat statistical program and the Mann–Whitney* U* test was used for data comparison with the level of significance set at *p* < 0.05.

## 3. Results

Over the treatment period, there were no signs of abnormalities or deaths. All groups exhibited a progressive increase in body weight as expected with growth (Table 1). Likewise, in Table 2, the progressive increase in red cell count, haemoglobin, and mean corpuscular haemoglobin concentrations (MCHC) over the 42 days of treatment, versus a decreased mean corpuscular volume (MCV) and mean corpuscular haemoglobin (MCH), was also observed in the control group, implying that it was also due to normal growth and development.

Table 3 shows that the renal and liver function tests were similar and within the normal range in all groups. In effect, there was no evidence of hepatotoxicity over the treatment period. Of note, the renal and liver function tests did not correlate with changes in the weight and FBC.

**Figure 1:**
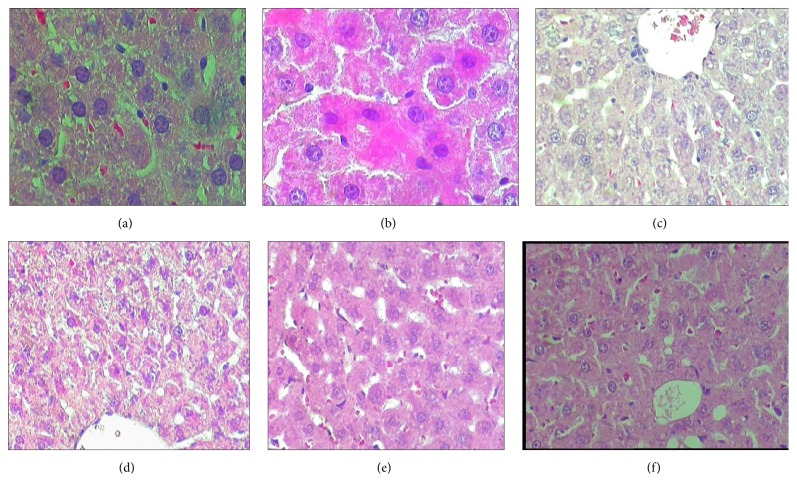
Representative histopathology slides (×20) of rat livers from control (untreated) and NVP treated rats and their respective pathology reports. (a) (No NVP): untreated group. “*No pathology appears to be present in the control (untreated) animals*”; (b) (NVP × 2 days): treated with NVP for 2 days. “*Mild granular vacuolar degeneration and cell swelling, scattered cytonecrosis, nuclear pyknosis. Zonal necrosis... minimally present.*”. (c) (NVP × 7 days): treated with NVP for 7 days. “*vacuolar degeneration and cell swelling were moderate, single cell necrosis (cytonecrosis)... minimal centrilobular necrosis.*”. (d) (NVP × 14 days): treated with NVP for 14 days. “*moderate cellular swelling, vacuolar hepatopathy (degeneration) and granular cytoplasm... Hepatic parenchymal cell necrosis (cytonecrosis), and centrilobular zonal necrosis*”. (e) (NVP × 28 days): treated with NVP for 28 days. “*Granular vacuolar degeneration and cell swelling were minimal, as well as cytonecrosis.*”. (f) (NVP × 42 days): treated with NVP for 42 days. “*Centrilobular hepatocytes with minimal (1+) degeneration visible*”.

**Figure 2:**
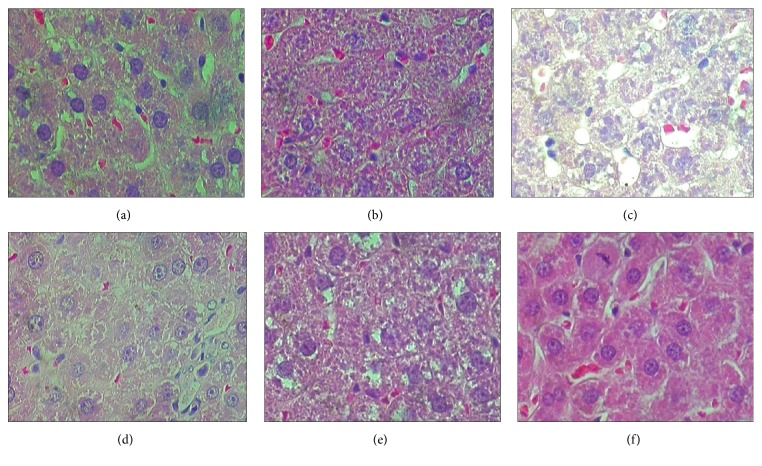
Representative histopathology slides (×20) of rat livers from control (untreated) and isoniazid (INH) treated rats and their respective pathology reports. (a) (No INH): untreated group. “*No pathology appears to be present in the control (untreated) animals*”; (b) (INH × 2 days): treated with INH for 2 days. “*Moderate granular vacuolar degeneration, loss of coordinated and well-organized hepatocytic cords. The cytoplasm appeared to be cloudy and granular, centrilobular zonal necrosis was minimal*”. (c) (INH × 7 days): treated with INH for 7 days. “*Severe degeneration of vacuoles and osmotic swelling of the mitochondria, cytonecrosis was moderate, loss of cell boundaries and nuclei*”. (d) (INH × 14 days): treated with INH for 14 days. “*Degenerative changes, cytonecrosis was present, disruption of the cytoplasm, minimal zonal necrosis was observed*”. (e) (INH × 28 days): treated with INH for 28 days. “*moderate cell swelling, mild cytonecrosis, hepatocytes appear swollen with granular cytoplasm.*”. (f) (INH × 42 days): treated with INH for 42 days. “*The histopathological lesions had improved, minimal cytonecrosis, centrilobular zonal necrosis was completely absent... one mitotic figure*”.

**Figure 3:**
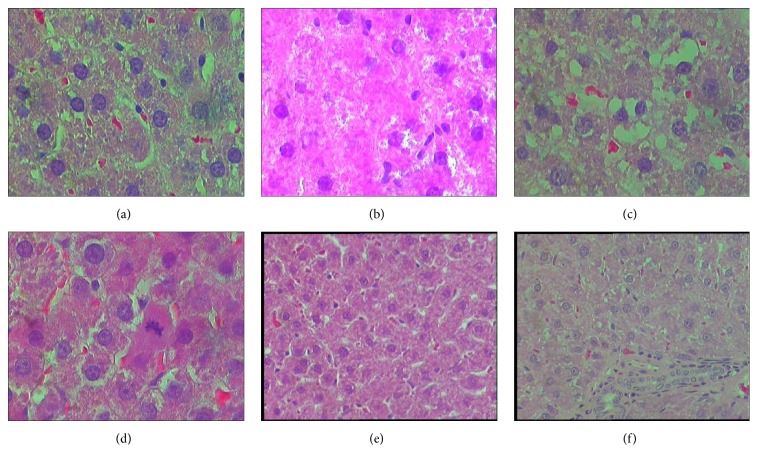
Representative histopathology slides (×20) of rat livers from control (untreated) and PAR treated rats and their respective pathology reports. (a) (No PAR): section of rat liver from the untreated group: “*No pathology appears to be present in the control (untreated) animals*”; (b) (PAR × 2 days): PAR for 2 days. “*moderate granular vacuolar degeneration and cell swelling, and minimal cytonecrosis, loss of cell boundaries, minimal mitosis, and minimal centrilobular zonal necrosis*”. (c) (PAR × 7 days): PAR for 7 days: “*mild degeneration and minimal cytonecrosis*”. (d) (PAR × 14 days): PAR for 14 days. “*moderate granular vacuolar degeneration and cell swelling, and minimal cytonecrosis loss of cell boundaries, minimal mitosis, and minimal hepatocyte mitosis*”. (e) (PAR × 28 days): PAR for 28 days: “*Hepatic cords with minimal vacuolar degenerative changes (×20)*”; (f) (PAR × 42 days): PAR for 42 days: “*Periportal hepatocytes, minimal degeneration visible (×20)*”.

Interestingly, contrary to the liver function tests, the histopathology changes exhibited evidence of hepatotoxicity in the first 28 days, followed by healing by day 42 (Figures 1, 2, and 3) for NVP, INH, and PAR, respectively. These figures show that the groups treated with NVP, INH and PAR exhibited abnormal liver histology within first 28 days and that the pathological lesion was similar. For NVP, the pathology lesions on days 2, 7, and 14 were reported as moderate degenerative changes such as vacuolar hepatopathy, cell swelling, and granular cytoplasm, with single cell necrosis (cytonecrosis) and minimal centrilobular zonal necrosis (Figures 1(b), 1(c), and 1(d)). By days 28 and 42, the lesions had improved and mitosis was present, an indicator of regeneration (Figures 1(e) and 1(f)). Likewise, INH induced liver pathology, though lasting longer up to day 28, was also described as moderate granular vacuolar degeneration and cell swelling with a cloudy and granular cytoplasm, as well as cytonecrosis and minimal centrilobular zonal necrosis (Figures 2(b), 2(c), 2(d), and 2(e)). By day 42, the lesions had improved, and mitosis was evident, indicating regeneration (Figure 2(f)). A similar observation was made for PAR but the liver injury lasted for 14 days (Figures 3(b), 3(c), 3(d), 3(e), and 3(f)). Of note, there was no liver cell necrosis or cellular infiltration, features commonly observed during overt hepatotoxicity.

**Figure 4:**
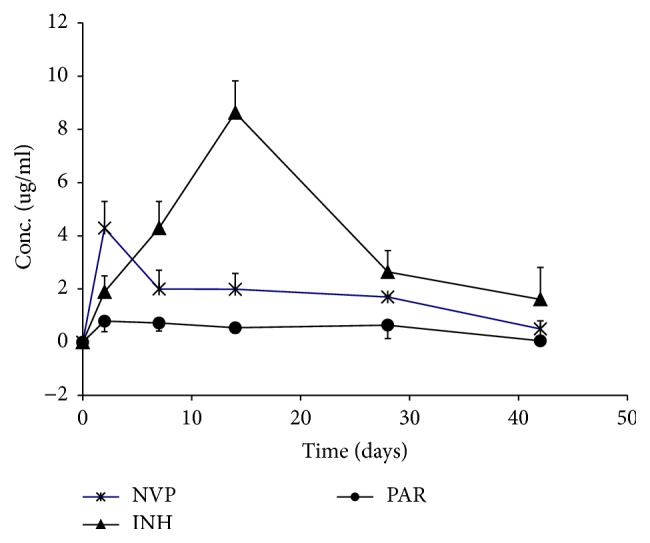
Plasma concentrations (mean ± sd) of NVP (stars), INH (triangles), and PAR (circles) in rats treated daily with NVP, INH, and PAR, respectively.

**Figure 5:**
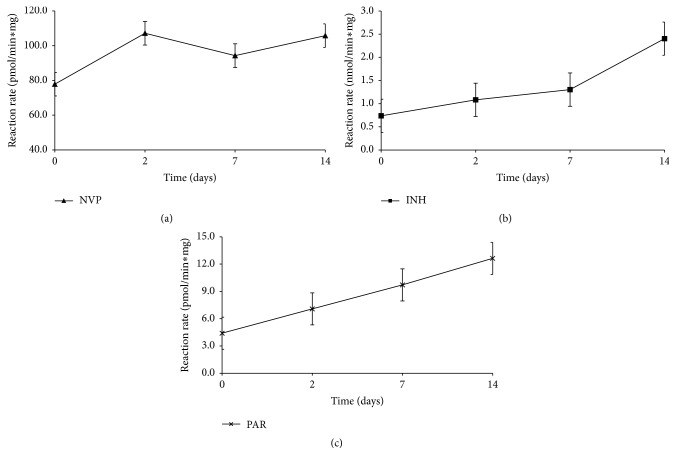
CYP3A2 activity in rats treated daily with NVP (a), CYP2E1 activity in rats treated daily with INH (b), and CYP1A2 activity in rats treated daily with PAR (c).

Figure 4 shows the plasma concentrations of NVP (Figure 4(a)), INH (Figure 4(b)), and PAR (Figure 4(c)) over the treatment period. Although all the respective drug concentrations were within the therapeutic ranges observed in humans, there was an association of increased drug concentration with the liver injury and subsequent low concentrations with the healing of the liver injury. This may also explain why, in the NVP and PAR groups, the liver injury healed faster (by day 14) owing to a faster drop in the NVP plasma concentrations, while the liver injury in the INH was longer (to 28 days) because INH concentrations peaked on day 7 and only dropped thereafter. Moreover, the fast drop in NVP concentration was associated with increased CYP3A2 activity (Figure 5(a)), the isoform responsible for NVP metabolism and activation [6], while for INH, there was increased CYP2E1 activity (Figure 5(b)) which is implicated in the hydrolysis of INH to hydrazine, a toxic metabolite [25, 26]. The PAR groups exhibited increased CYP1A2 activity (Figure 5(c)), one of the isoforms implicated in activation of PAR [10].

**Figure 6:**
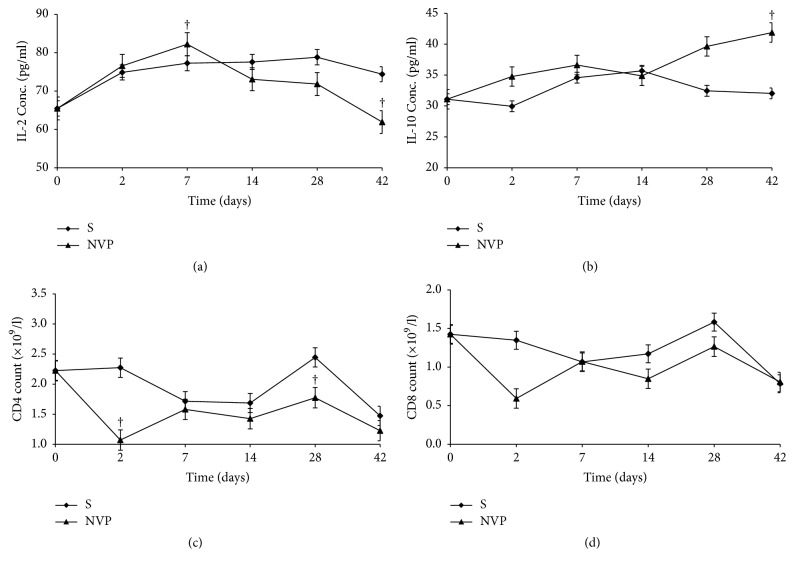
Serum concentrations (mean ± sd) of IL-2 (a), IL-10 (b), and CD4 (c) and CD8 (d) count during treatment with nevirapine (NVP) and saline (S). Note: † = *p* ≤ 0.05.

**Figure 7:**
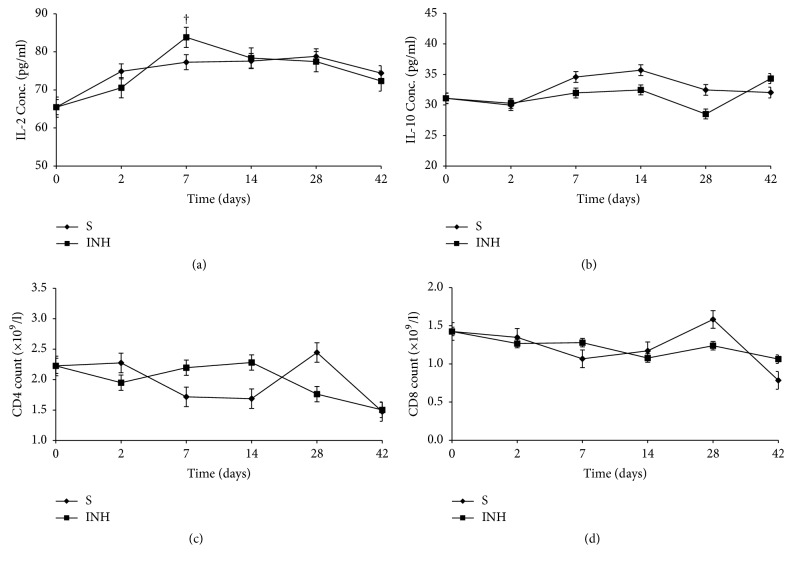
Serum concentrations (mean ± sd) of IL-2 (a), IL-10 (b), and CD4 (c) and CD8 (d) count during treatment with isoniazid (INH) and saline (S). Note: † = *p* ≤ 0.05.

**Figure 8:**
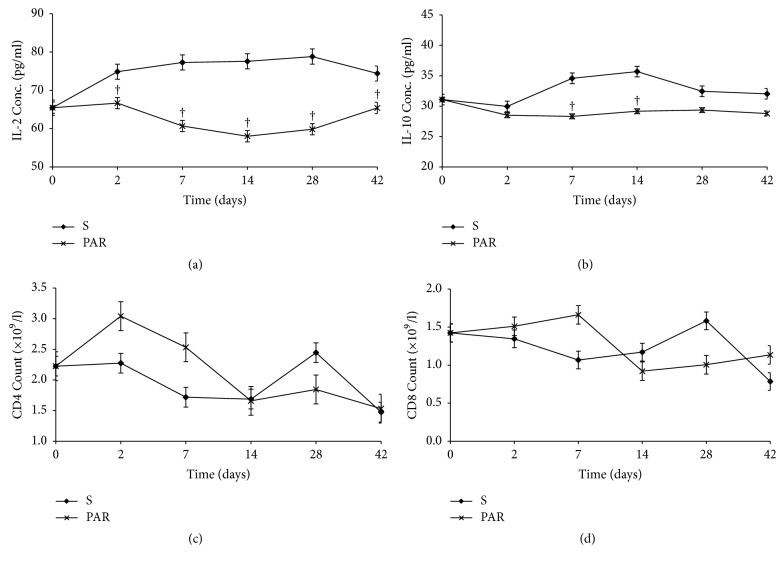
Serum concentrations (mean ± sd) of IL-2 (a), IL-10 (b), and CD4 (c) and CD8 (d) count during treatment with paracetamol (PAR) and saline (S). Note: † = *p* ≤ 0.05.

Figure 6 shows the corresponding changes in the immune parameters in for the NVP treated group. IL-2 increased from day 2 to day 7 and then dropped progressively up to day 42 (Figure 6(a)), while IL-10 increased from day 14 to day 42 (Figure 6(b)). The changes in CD4 and CD8 counts were not different from the control (Figures 6(c) and 6(d)). Treatment with INH led to increased IL-2 by day 7 (Figure 7(a)), which was associated with a relatively higher CD4 count on days 7 and 8 (Figure 7(c)), while IL-10 levels and CD8 count were not different from the control. This implies that the changes in IL-2 were not strong enough to induce a change in IL-10 and CD8. PAR treatment led to lower IL-2 and IL-10 levels than in the control (Figures 8(a) and 8(b)) versus a moderately increased CD4 and CD8 counts on days 2 and 7 (Figures 8(c) and 8(d)). Whereas the cause for lower cytokine levels is not clear, the changes in CD4 and CD8 counts imply that there was probably antigen dependent activation of these cells, but this dissipated by day 14 owing to lack of propagation usually by IL-2.

## 4. Discussion

This study has demonstrated the initial subclinical drug-induced liver injury and its subsequent healing in association with the adaptive immune response, thereby confirming the postulations by Metushi et al., 2014 and 2016 [16, 17]. The subclinical pathological changes in the liver were compared with the immune response and activity of some CYP450 enzymes during prolonged daily administration of the three well known hepatotoxic drugs at normal doses in animals that did not develop clinical hepatotoxicity. Of note, clinical hepatotoxicity is when the increase in liver enzymes, alanine transferase (ALT) and aspartate transferase (AST), is 3–5 times the upper limit of the normal range [27], and this departure from the normal range may be confirmed by comparative statistical testing and drug-induced characteristic pathology lesions in the liver. In this study, the changes described for the respective parameters occurred within their normal concentration ranges; that is, the liver injury was subclinical and concentrations of the cytokines and the drugs were within their normal ranges. These early changes were studied on the premise that they are the premonitory symptoms of a major pathological process; that during normal dosing, the body systems, which include the immune system and CYP450 enzymes, are able to respond to drug insults as indicated by increased or decreased cytokines or enzyme activity but within their normal range; and that understanding these early changes is vital not only to unveiling the mechanism of the respective drug-induced hepatotoxicity but also for development of preventive strategies. In the same perspective, for this subclinical state, the changes or responses in the test group relative to those in the control may not need to be statistically significant to be useful.

The changes in the two cytokines studied (IL-2 and IL-10) were more classical with NVP treatment where increased IL-2 was observed in the first 7 days, denoting a Th1 response, and was associated with liver injury. Later, this shifted to increased IL-10, denoting a Th2 response, and was associated with healing of the liver injury. The associated lower CD4 and CD8 counts were probably due to the counterregulatory effects of the Th2 response as indicated by the increased IL-10. This would prevent the effects of IL-2 such as stimulating further proliferation of CD4 and CD8.

The changes in the profiles of the two cytokines were not so marked in the INH and PAR treated groups. For INH, there was no difference from the control, which implies that the changes in IL-2 were not strong enough to induce a change in IL-10 and CD8. Whereas the cause for lower cytokine levels in the PAR treated groups was not clear, the mild increases in CD4 and CD8 counts on days 2 to 7 imply that there was probably antigen dependent activation of these cells, but this dissipated by day 14 owing to lack of propagation usually by IL-2. In effect, there was immune stimulation though at a lower degree compared to NVP response. Indeed, this observation with the two cytokines does not mean that there was no immune response in the INH and PAR groups because there are other immune markers not analysed, which could have perhaps mediated the liver injury [10, 11]. Most important is that it could point to differences in the intricate mechanisms by which each of the drugs interacts with the immune system.

Here, IL-2 and IL-10 were selected based on our experience in the previous studies with NVP [20] and clinical reports where reduced expression of IL-10 [28, 29] and neutralisation of IL-10 in mice with antibodies [15, 30] were associated with development of hepatotoxicity. Of note, IL-2 and IL-10 are among the earliest produced cytokines with distinct and consistent roles in the primary immune response. During the primary immune response, IL-2 stimulates proliferation of many immune cells (CD4, CD8, and macrophages) as well as the production of several important cytokines such as IL-4, IFN-*γ*, and TGF-*β*, while IL-10 is a strong counterregulatory cytokine that suppresses Th1-cytokine induced inflammation [31–33]. As such, our laboratory has perfected the assays for these two cytokines and used them as primers for decisions regarding the need to analyse other markers of the immune system.

However, the fact that the liver pathological lesions were similar for each of the three drugs implies that the immune system is responsible for the initial insult and alludes to why most drug-induced hepatotoxicity, excluding that due to overdose, is not distinguishable by pathological lesions or evaluations. Furthermore, contrary to the common assumption that involvement of the immune system would be exhibited by inflammation, there was no evidence of inflammation in the liver tissue at this early stage (see pathology report). This means that overt inflammation and the disruption of liver function tests enzymes are late stage processes depending on the progress of the liver injury. But most importantly here, it indicates that the initial immune responses are well coordinated, purposeful, and target specific. Therefore, seeking for evidence of general inflammatory response or hypersensitivity reaction would not be appropriate.

The close relationship between the drug concentrations and the liver injury implies that the respective drugs are still major contributors to the liver injury and further confirms that, despite the earlier reports on the noninvolvement of CYP450 in NVP-induced hepatotoxicity [34], the drug metabolising enzymes are in some way involved. Specifically, the rapid fall in NVP and PAR concentrations was associated with an early resolution of the liver injury after day 14, while high INH concentrations were associated with a late resolution of the liver injury to 28 days. This concentration relationship could be due to differences in the implications of increased enzyme activity, as well as in the structural aspects of the individual drug molecules. Changes in enzyme activity may lead to increased drug elimination hence, faster removal of the offending drug, or metabolic activation leading to increased toxic products. Here, the increased activity of CYP3A in the NVP group was probably responsible for fast elimination of NVP, while for INH, increased CYP2E1 activity (the isoform implicated in the hydrolysis of INH to hydrazine, a toxic metabolite) was most probably responsible for the persistent liver injury [26, 35, 36]. Of note, INH is an inducer of CYP2E1. For PAR, the concentrations were within the body's metabolic capacity; as such, increased CYP1A2 did not lead to increased liver injury.

Regarding structural aspects, the size and complexity of the antigen can determine the degree of immune response whereby intermediate size antigens or complexes are better recognised and therefore more immunogenic than small ones [37]. In this case, compared to INH and PAR, NVP has the biggest structure of a three-ring reactive molecule with a M.wt of 266.3, versus the single ring INH (M.wt. 137.1) and PAR (M.wt. 151.2). Therefore, the bigger adducts such as those for NVP are more immunogenic than the smaller adducts of INH and PAR; as such, NVP elicited a classical Th1/2 immune response. In effect, this suggests that the immune clearing mechanism of apoptosis was more enhanced in the NVP group, leading to a faster resolution of the liver injury. Unfortunately, for INH, it is activated to several reactive metabolites at different times in the metabolic pathway, but all these metabolites are small in size. The earliest immunogenic products would be the small adducts with hydrazine, a reactive metabolite produced by direct hydrolysis of isoniazid, and later from the acetylhydrazine and acetylisoniazid [26, 35, 36]. This could account for the poor immune response via IL-2 and IL-10 and the slower clearing mechanism of apoptosis versus continuous insult of liver to 28 days.

Since the time of resolution of the liver injury in these animals that did not develop clinical hepatotoxicity marks the time at which the immune system has successfully adapted to the drug, it would also mark the time for the start of progression to clinical hepatotoxicity in those where the adaptive mechanisms fail. In fact, the times taken for the resolution of the NVP and INH liver injury observed in this study are similar to the time of onset of clinical hepatotoxicity by both drugs in patients, that is, 2–4 weeks for NVP and 1–3 months for INH. This implies that this animal model mimics the clinical situation and therefore provides an opportunity for further studies on these subclinical changes in order to determine the mechanism and factors influencing drug-induced hepatotoxicity. It calls for a comprehensive approach in evaluating drug-induced hepatotoxicity by simultaneous monitoring of the sequence of response by all relevant cellular signals (molecular mechanisms), immune or metabolic, while, appreciating the limitations of each mechanism, it is unlikely that one mechanism will be the answer to everything.

Although PAR is commonly associated with acute hepatotoxicity after overdose, there are several reports of PA-induced hepatotoxicity during chronic dosing at therapeutic doses (≤4 g/day) in patients without known predisposing factors [38]. Since INH and NVP are commonly used in combination with other drugs, further studies are needed for the respective combinations.

Overall, all the three drugs induced a subclinical liver injury in the first 2-3 weeks followed by healing. The liver injury was pathologically similar and was associated with immune stimulation and increased cytochrome P450 activity.

In conclusion, the initial subclinical drug-induced liver injury and its healing in association with the adaptive immune response have been demonstrated.

## Figures and Tables

**Table 1:** Change in body weight (mean ± SD) during treatment of the rats with NVP, INH, and PAR over the study period.

Days of treatment	Change in weight (g)			
	S	NVP	INH	PAR
2 days	9.2 ± 4	10.6 ± 8	7.0 ± 6	9.7 ± 6
7 days	35.6 ± 8	28.5 ± 1	31.0 ± 19	34.6 ± 4
14 days	84.6 ± 5	82.8 ± 2	55.0 ± 10	62.6 ± 13
28 days	107.8 ± 10	99.7 ± 1	111.9 ± 12	103.5 ± 14
42 days	171.4 ± 27	105.6 ± 16	141.3 ± 14	147.5 ± 20

S: saline; INH: isoniazid; NVP: nevirapine; PAR: paracetamol; *p* ≤ 0.05.

**Table 2:** Average (mean ± SD) full blood count and platelets during treatment of the rats with NVP, INH, and PAR over the study period.

Group	RCC (×10^12^/l)	Hb (g/dl)	Hct (l/l)	MCV (fl)	MCH (pg)	MCHC (g/dl)	Plt (×10^9^/l)	WCC (×10^9^/l)	Neu (×10^9^/l)	Ly (×10^9^/l)	Mo (×10^9^/l)	Eos (×10^9^/l)	Bas (×10^9^/l)
*Untreated*													
0 days	6.28 ± 0.2	12.9 ± 0.3	0.398 ± 0.01	63.5 ± 2.5	20.5 ± 0.4	32.3 ± 0.8	860 ± 221.1	6.95 ± 2.7	0.77 ± 0.2	4.67 ± 1.8	0.19 ± 0.1	0.02 ± 0.0	0.01 ± 0.0
*S*													
2 days	6.67 ± 0.2	13.7 ± 0.1	0.422 ± 0.01	63.3 ± 2.3	20.6 ± 0.6	32.4 ± 0.6	849 ± 81.6	6.50 ± 0.9	0.60 ± 0.2	5.18 ± 0.7	0.21 ± 0.0	0.50 ± 0.2	0.01 ± 0.0
7 days	7.53 ± 0.9	15.3 ± 1.7	0.451 ± 0.04	60.1 ± 2.5	20.3 ± 0.2	33.9 ± 1.2	1033 ± 79.8	5.44 ± 2.4	1.03 ± 0.8	4.07 ± 2.0	0.30 ± 0.3	0.04 ± 0.0	0.01 ± 0.0
14 days	6.77 ± 0.6	13.9 ± 1.1	0.417 ± 0.03	61.8 ± 2.8	20.5 ± 0.6	33.2 ± 0.6	721 ± 196.4	5.22 ± 1.2	0.63 ± 0.5	4.21 ± 0.7	0.18 ± 0.1	0.18 ± 0.1	0.05 ± 0.0
28 days	7.07 ± 0.7	13.9 ± 1.3	0.390 ± 0.04	55.1 ± 1.0	19.7 ± 0.1	35.8 ± 0.6	961 ± 172.5	7.38 ± 1.0	0.91 ± 0.2	6.15 ± 0.8	0.24 ± 0.1	0.07 ± 0.0	0.01 ± 0.0
42 days	6.93 ± 0.8	13.4 ± 1.8	0.374 ± 0.05	53.9 ± 1.0	19.3 ± 0.4	35.8 ± 0.2	839 ± 166.0	3.93 ± 0.3	0.54 ± 0.1	3.23 ± 0.3	0.11 ± 0.0	0.04 ± 0.0	0.01 ± 0.0
*NVP*													
2 days	6.51 ± 0.2	13.6 ± 0.4	0.408 ± 0.01	62.7 ± 0.8	20.9 ± 0.1	33.2 ± 0.3	854 ± 209.3	3.48 ± 0.8	1.02 ± 0.1	1.88 ± 0.3	0.10 ± 0.0	0.48 ± 0.4	0.00 ± 0.0
7 days	7.14 ± 0.3	14.2 ± 0.5	0.424 ± 0.02	59.5 ± 1.8	19.9 ± 0.6	33.5 ± 0.5	779 ± 141.3	5.54 ± 1.3	0.76 ± 0.3	3.81 ± 0.9	0.49 ± 0.2	0.48 ± 0.2	0.03 ± 0.0
14 days	6.97 ± 0.3	13.9 ± 0.4	0.415 ± 0.01	59.5 ± 2.2	19.9 ± 0.6	33.5 ± 0.3	1013 ± 73.0	4.86 ± 0.7	1.08 ± 0.2	3.38 ± 0.4	0.37 ± 0.1	0.02 ± 0.0	0.01 ± 0.0
28 days	7.36 ± 0.6	14.2 ± 1.1	0.404 ± 0.03	55.0 ± 0.7	19.3 ± 0.3	35.0 ± 0.6	876 ± 17.2	6.07 ± 0.6	1.09 ± 0.2	4.62 ± 0.7	0.28 ± 0.2	0.08 ± 0.1	0.01 ± 0.0
42 days	7.73 ± 0.3	14.5 ± 0.3	0.405 ± 0.00	52.5 ± 2.3	18.8 ± 0.5	35.8 ± 0.8	1052 ± 115.6	5.42 ± 0.1	1.52 ± 0.6	3.52 ± 0.6	0.33 ± 0.0	0.04 ± 0.0	0.01 ± 0.0
*INH*													
2 days	6.57 ± 0.3	13.4 ± 0.4	0.416 ± 0.01	63.4 ± 2.0	20.5 ± 0.3	32.3 ± 0.7	880 ± 54.5	5.86 ± 1.1	0.59 ± 0.0	5.02 ± 1.1	0.21 ± 0.1	0.03 ± 0.0	0.01 ± 0.0
7 days	6.05 ± 0.6	12.7 ± 1.1	0.381 ± 0.04	63.0 ± 0.6	21.0 ± 0.2	33.3 ± 0.2	478 ± 19.1	5.98 ± 0.8	0.56 ± 0.1	5.10 ± 0.8	0.19 ± 0.1	0.04 ± 0.0	0.02 ± 0.0
14 days	6.65 ± 0.6	9.8 ± 5.4	0.394 ± 0.04	59.2 ± 2.7	19.8 ± 0.6	33.4 ± 0.7	494 ± 50.2	5.78 ± 1.1	0.61 ± 0.1	4.91 ± 0.9	0.23 ± 0.1	0.03 ± 0.0	0.00 ± 0.0
28 days	7.27 ± 0.4	14.3 ± 0.8	0.406 ± 0.02	55.9 ± 0.6	19.7 ± 0.1	35.3 ± 0.4	845 ± 116.0	5.44 ± 0.4	0.66 ± 0.1	4.56 ± 0.4	0.17 ± 0.1	0.04 ± 0.0	0.01 ± 0.0
42 days	7.25 ± 0.7	13.9 ± 1.2	0.390 ± 0.04	53.8 ± 1.9	19.2 ± 0.4	35.6 ± 0.8	785 ± 152.7	5.11 ± 0.9	0.84 ± 0.1	4.05 ± 0.6	0.22 ± 0.0	0.07 ± 0.0	0.01 ± 0.0
*PAR*													
2 days	6.77 ± 0.0	14.8 ± 0.0	0.461 ± 0.00	68.1 ± 0.0	21.9 ± 0.0	32.1 ± 0.0	419 ± 0.0	7.54 ± 0.0	0.75 ± 0.0	5.96 ± 0.0	0.37 ± 0.0	0.46 ± 0.0	0.01 ± 0.0
7 days	6.75 ± 0.2	13.9 ± 0.1	0.426 ± 0.00	63.2 ± 2.4	20.7 ± 0.8	32.7 ± 0.1	807 ± 22.8	7.32 ± 0.8	0.80 ± 0.2	6.22 ± 0.8	0.23 ± 0.0	0.07 ± 0.0	0.01 ± 0.0
14 days	6.66 ± 0.2	13.8 ± 0.3	0.411 ± 0.01	61.7 ± 0.5	20.7 ± 0.1	33.6 ± 0.4	834 ± 43.0	4.19 ± 0.4	0.55 ± 0.0	3.42 ± 0.6	0.09 ± 0.1	0.13 ± 0.2	0.00 ± 0.0
28 days	7.10 ± 0.2	14.1 ± 0.2	0.397 ± 0.00	56.0 ± 2.2	19.8 ± 0.8	35.4 ± 0.4	880 ± 87.0	5.32 ± 1.2	0.88 ± 0.1	4.19 ± 1.1	0.21 ± 0.1	0.04 ± 0.0	0.01 ± 0.0
42 days	7.33 ± 0.3	14.5 ± 0.3	0.401 ± 0.01	54.8 ± 1.8	19.8 ± 0.5	36.2 ± 0.3	961 ± 85.8	4.91 ± 0.3	0.80 ± 0.2	3.86 ± 0.4	0.20 ± 0.0	0.05 ± 0.0	0.01 ± 0.0

RCC: red cell count; Hb: haemoglobin; Hct: haematocrit; MCV: mean corpuscular volume; MCH: mean corpuscular haemoglobin; MCHC: mean corpuscular haemoglobin concentration; Plt: platelets; WCC: white cell count; Neu: neutrophils; Ly: lymphocytes; Mo: monocytes; Eos: eosinophils; Bas: basophils, S: saline; INH: isoniazid; NVP: nevirapine; PAR: paracetamol; *p* ≤ 0.05.

**Table 3:** Average (mean ± SD) change in renal and liver function tests during treatment of the rats with NVP, INH, and PAR over the study period.

Group	RFT		LFT		
	BUN (mmol/l)	Cr (*µ*mol/l)	ALT (U/l)	AST (U/l)	ALP (U/l)
*Untreated*					
0 days	7.2 ± 1	37 ± 8	50 ± 5	88 ± 14	352 ± 76
*S*					
2 days	7.3 ± 1	39 ± 2	46 ± 2	90 ± 7	400 ± 7
7 days	8.1 ± 0	46 ± 7	49 ± 1	103 ± 25	304 ± 13
14 days	7.5 ± 1	39 ± 3	58 ± 4	127 ± 37	508 ± 37
28 days	10.6 ± 2	73 ± 2	47 ± 2	115 ± 44	216 ± 19
42 days	5.8 ± 1	38 ± 9	46 ± 6	76 ± 28	109 ± 76
*NVP*					
2 days	7.9 ± 1	36 ± 1	63 ± 7	107 ± 10	359 ± 43
7 days	8.7 ± 0	46 ± 2	87 ± 4	169 ± 15	47 ± 78
14 days	9.1 ± 1	41 ± 9	72 ± 3	109 ± 33	443 ± 43
28 days	8.5 ± 1	63 ± 7	53 ± 4	128 ± 44	166 ± 37
42 days	7.0 ± 0	27 ± 3	54 ± 2	70 ± 4	14 ± 9
*INH*					
2 days	6.7 ± 0	34 ± 6	46 ± 4	104 ± 7	335 ± 32
7 days	6.6 ± 0	36 ± 2	53 ± 1	233 ± 22	369 ± 9
14 days	6.2 ± 1	43 ± 8	43 ± 4	98 ± 29	364 ± 28
28 days	7.3 ± 1	69 ± 4	46 ± 4	143 ± 36	220 ± 29
42 days	5.8 ± 0	34 ± 2	51 ± 2	86 ± 14	127 ± 78
*PAR*					
2 days	6.7 ± 1	36 ± 6	54 ± 4	109 ± 6	402 ± 64
7 days	7.8 ± 1	37 ± 5	58 ± 2	104 ± 30	349 ± 27
14 days	7.3 ± 1	36 ± 2	53 ± 2	95 ± 12	364 ± 38
28 days	7.3 ± 1	73 ± 4	49 ± 4	122 ± 35	224 ± 46
42 days	6.9 ± 1	24 ± 5	48 ± 5	64 ± 3	55 ± 64

RFT: renal function test; BUN: blood urea nitrogen; Cr: creatinine; LFT: liver function test; ALT: alanine aminotransferase; AST: aspartate aminotransferase; ALP: alkaline phosphatase; S: saline; INH: isoniazid; NVP: nevirapine; PAR: paracetamol; *p* ≤ 0.05.
